# Therapeutic Migration in Interstitial Cystitis/Bladder Pain Syndrome: Cross-Organ Lessons for Urothelial Healing and Endotype-Guided Therapy

**DOI:** 10.3390/biomedicines14102266

**Published:** 2026-10-07

**Authors:** Jianguo Wang, Ning Zhang, Shiyao Yang, Weiguo Chen

**Affiliations:** Department of Urology, The First Affiliated Hospital of Soochow University, Suzhou 215006, China; 15647998730@163.com (J.W.); zhangning250909@163.com (N.Z.); adyangshiyao@163.com (S.Y.)

**Keywords:** interstitial cystitis, bladder pain syndrome, therapeutic migration, urothelial healing, endotype, treat-to-target, regenerative medicine, microbiome

## Abstract

Interstitial cystitis/bladder pain syndrome (IC/BPS) is biologically heterogeneous. No single bladder-directed therapy addresses every relevant mechanism. Recent reviews have covered pathophysiology, multimodal care, and biomaterial-based mucosal repair in detail. This review takes a different route and uses therapeutic migration as a translational lens. The concept refers to moving a treatment idea from another specialty into IC/BPS when the same biological problem appears to be active in a bladder endotype. Representative migrations include platelet-rich plasma and cell-based repair strategies, along with neural, immune-directed, and microbiome approaches. We also discuss one unsuccessful migration. The central question is whether the biological rationale still holds in the bladder. We therefore propose a migration filter that considers mechanism conservation, endotype enrichment, feasibility in the urinary environment, safety, and objective target engagement. Urothelial healing is discussed as a tissue-recovery construct for bladder-centered phenotypes, while treat-to-target principles are adapted for mechanism-informed research. Therapeutic migration remains the overarching framework, but symptom control and biological recovery are treated as separate outcomes.

## 1. Introduction

IC/BPS remains difficult to classify and treat because it is not a single biological entity. Recent guideline and consensus work increasingly separates Hunner lesion disease from non-Hunner presentations [1,2,3,4]. The former is typically more bladder-centered and inflammatory. The latter can include subtler urothelial abnormalities, but pain may also be shaped by pelvic-floor dysfunction, widespread pain, autonomic features, or central sensitization. This heterogeneity helps explain why symptom-based treatment often produces variable and modest effects [2,5,6].

Urothelial barrier dysfunction provides a useful disease model, but it does not explain every case. Surface protection depends on the glycosaminoglycan (GAG)/glycocalyx layer and differentiated umbrella cells. Junctional complexes and regenerative capacity help maintain integrity, while an underlying immune, neural, and vascular compartment shapes how injury is sensed and amplified. When this system is disturbed, urinary solutes and danger signals can gain greater access to the lamina propria and intensify inflammatory or nociceptive signaling [7,8,9,10]. In some patients, barrier injury may be secondary rather than initiating. It can still become an important amplifier.

Several recent reviews already summarize IC/BPS pathophysiology, multimodal treatment, and current bladder therapies [6,10]. A 2026 review also focuses on biomaterials, nanomedicine, mucosal repair, and drug-delivery strategies [11]. Our question is different. We ask how treatment concepts move across organ systems, why a migration may succeed in one IC/BPS endotype and fail in another, and how mechanism-specific outcomes can be used to judge that transfer. Urothelial-healing measures are included when they are biologically appropriate. Drug-delivery engineering is discussed only when exposure changes interpretation of the target.

Therapeutic migration, as used here, transfers a treatment principle between diseases or organs when its biological premise can be tested in the bladder. It is one route within translational medicine. Drug repurposing may be part of that route, but the examples in this review also include procedures, cells, biologics, and microbial interventions. The key questions are whether the proposed target exists in a defined IC/BPS group and what outcomes would support or refute the transfer. The framework has not been validated for choosing treatment in current practice.

## 2. Review Approach and Scope

This is a narrative, hypothesis-generating review rather than a systematic review or clinical guideline. Candidate publications were identified through targeted searches of PubMed/MEDLINE and publisher full-text sites, citation tracking from relevant guidelines and primary studies, and review of regulatory safety documents where applicable. The final targeted update was performed on 1 October 2026. Principal search concepts included “interstitial cystitis” OR “bladder pain syndrome”, combined with “Hunner lesion”, “urothelial barrier”, “endotype” OR “phenotype”, “mucosal healing”, “platelet-rich plasma”, “mesenchymal stem cell”, “neuromodulation”, “botulinum toxin”, “microbiota transplantation”, and “bacillus Calmette–Guérin”. Cross-organ searches paired “inflammatory bowel disease”, “oral mucositis”, “chronic wound”, “eosinophilic esophagitis”, “chronic rhinosinusitis”, “vasculitis”, “radiation cystitis”, or “recurrent urinary tract infection” with barrier, immune, repair, or treat-to-target concepts. English- and Chinese-language publications were considered; other languages were not systematically searched.

Comparator diseases were chosen because they pose a biological or practical question relevant to IC/BPS. We traced interventions to other specialties, examined shared processes such as barrier injury, inflammation, neural sensitization, and impaired repair, and considered the constraints of local treatment in luminal tissues. Clinical associations with systemic disease were treated as a prompt for inquiry only when a common process was plausible. IBD informs mucosal assessment and treat-to-target design; oral mucositis and chronic wounds illuminate injury and restitution; eosinophilic esophagitis and chronic rhinosinusitis offer examples of pathway-defined therapy. Vasculitis provides an immune–vascular comparison, while radiation cystitis and recurrent UTI test assumptions about treatment in the urinary environment. We favored guidelines, controlled or prospective studies, and primary human or mechanistic work, while retaining pivotal older trials and negative findings. Selection was purposive, not exhaustive. We did not duplicate screening, register a protocol, formally grade risk of bias, pool effects, or apply GRADE. Cross-organ findings remain hypotheses unless the relevant process is shown in bladder tissue or a defined IC/BPS population.

## 3. Urothelial Barrier Dysfunction in IC/BPS: Established Biology and Heterogeneity

### 3.1. Barrier Architecture, Sensory Signaling, and Repair

The urothelium is not an inert lining. Its GAG/glycocalyx layer limits direct contact between urine and the cell surface, while umbrella-cell uroplakin plaques create a highly impermeable but mechanically adaptable membrane. Tight-junction and adherens-junction proteins restrict paracellular flux. Urothelial cells also respond to stretch and chemical changes. Through released mediators, they communicate with immune cells, afferent nerves, stromal cells, and detrusor muscle [7,8,10].

A clinically useful barrier model should not be reduced to one defect. Surface protection and intercellular sealing are only part of the problem. Sensory signaling may remain abnormal even when the luminal surface is partly restored, and defective repair can persist after inflammation has fallen. A therapy that coats the bladder may therefore improve one layer of dysfunction while leaving another largely unchanged.

### 3.2. The Barrier–Amplification Loop

Barrier failure increases contact between the suburothelium and urinary irritants. Resident immune pathways may then become activated, and afferent excitability can rise. Oxidative stress can intensify the process. Reactive oxygen species (ROS) can disrupt membranes, mitochondria, and junctional localization [9]. Inflammatory recruitment and tissue hypoxia add further oxidative burden. Adenosine triphosphate (ATP), nerve growth factor (NGF), and transient receptor potential (TRP) signaling can then lower sensory thresholds and contribute to pain, urgency, and frequency.

**Figure 1:**
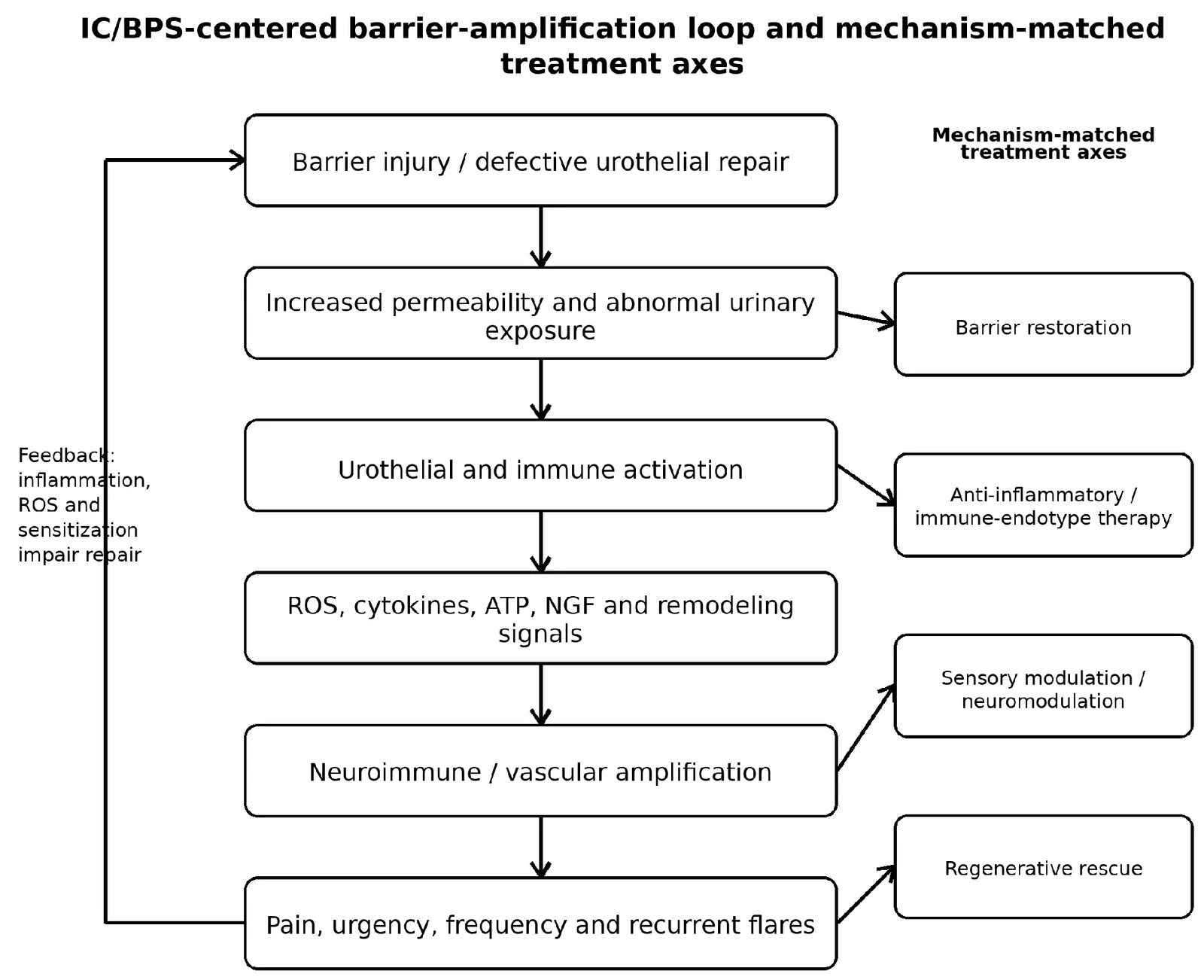
IC/BPS-centered barrier–amplification loop and mechanism-matched treatment axes. Barrier dysfunction can interact with immune activation, oxidative stress, neurogenic amplification, microvascular change, and defective repair. The relative importance and directionality of these components may differ among phenotypes; the figure does not imply that barrier injury initiates all IC/BPS.

Once this loop is established, it may become partly self-sustaining. Abnormal afferent activity can persist. So can microvascular dysfunction or incomplete epithelial repair. These processes may keep the barrier vulnerable after the initiating trigger has faded. The model is compatible with recurrent flares, but it should not be treated as a universal causal sequence. Figure 1 summarizes the proposed amplification loop and the corresponding treatment axes.

### 3.3. Hunner Lesion Disease and Non-Hunner Phenotypes

Recent histopathological and genomic studies further separate Hunner lesion disease from non-Hunner IC/BPS. Hunner lesions are associated with urothelial denudation and chronic subepithelial inflammation; lymphoplasmacytic infiltration, vascular change, and fibrosis are also described [4,12]. Immunogenomic profiling has implicated clonally expanded B cells and signaling through B-cell activating factor (BAFF) and a proliferation-inducing ligand (APRIL) [4,12]. Non-Hunner disease often preserves the urothelial layers and shows subtler or more heterogeneous inflammatory changes [4]. These differences argue against merging bladder-centered inflammatory disease with systemic or myofascial pain phenotypes.

The 2025 global consensus phenotyping effort recommends, at minimum, separating bladder-centric from systemic presentations [3]. That distinction matters in a barrier-oriented model. Objective urothelial injury is more likely to be a primary treatment target in bladder-centric disease. In a systemic-pain phenotype, it may be only one part of a wider pain network.

### 3.4. Biomarkers, Microbiota, and the Limits of Single-Marker Diagnosis

Urinary and serum biomarker studies have identified signals related to inflammation, oxidative stress, neurotrophins, and extracellular-matrix remodeling [13,14,15]. Some differ between IC/BPS and controls; others vary across clinical phenotypes. Matched serum-urine analyses and machine-learning studies suggest that multianalyte panels may outperform individual markers for phenotype or response prediction [13,16,17]. Recent reviews have therefore shifted attention away from a single diagnostic molecule and toward biomarker clusters [14,15,17].

Microbiome studies add another source of heterogeneity [18]. Their results are sensitive to specimen collection and contamination control, and they are also influenced by antibiotic exposure, hormonal status, and phenotyping. The microbiota are therefore better viewed as a possible modifier than as evidence of chronic occult infection.

Mechanistic work is beginning to move beyond association. A recent multi-omics study of Hunner-type IC/BPS combined metagenomics, bile-acid metabolomics, and single-cell profiling. It identified an axis linking *Enterococcus avium*, taurochenodeoxycholic acid (TCDCA), and TLR3 to urothelial injury. Transfer and metabolite experiments reproduced barrier disruption, whereas TLR3 inhibition attenuated injury [19]. These findings do not show that microbiota transplantation is effective in IC/BPS. They do, however, provide a mechanistic route by which dysbiosis could influence the bladder through defined metabolites and urothelial signaling.

## 4. Cross-Organ Mechanistic Convergence: What Is Actually Transferable?

Cross-organ comparison is useful when it identifies a biological problem that can also be measured in the bladder. The comparator diseases in this review are not treated as equivalents of IC/BPS. Instead, they provide models for asking whether barrier failure, immune activation, neural sensitization, defective repair, or another relevant process is conserved enough to justify a bladder-specific hypothesis. A shared pathway, clinical association, or delivery problem is therefore a reason to test transfer, not evidence that the transfer will succeed.

### 4.1. Barrier Failure, Repair Failure, and Recurrence

Inflammatory bowel disease (IBD) is the strongest structural comparator because both the intestine and bladder face a complex luminal environment. In the intestine, epithelial differentiation and junctional regulation work together with mucus and antimicrobial defenses to maintain barrier function. When that barrier fails, antigen access increases and immune activation follows. Inflammation, in turn, impairs junctional regulation and epithelial repair [20,21].

The oral mucosa offers a different kind of comparison. It is a high-turnover barrier exposed to repeated mechanical and chemical stress. Therapy-associated oral mucositis is useful because injury can be followed from epithelial damage through inflammation and pain into repair [22,23,24]. IC/BPS is not oral mucositis, but this sequence provides a tractable model for studying how tissue injury and nociception interact.

Chronic wounds make repair failure experimentally visible. Many remain in a prolonged inflammatory state, with oxidative stress and impaired cellular migration limiting re-epithelialization. Randomized wound-healing studies provide direct evidence that autologous PRP can accelerate closure of selected chronic diabetic foot ulcers [25]. For a repair-deficient IC/BPS phenotype, the question is more specific: can platelet-derived repair signals be delivered to the bladder in a way that helps epithelial recovery without adding fibrosis, inflammation, or procedural injury?

Same-organ comparators help prevent overinterpretation of distant analogies. Radiation cystitis combines urothelial injury with inflammation, vascular damage, and fibrosis [26]. In IC/BPS itself, a randomized multicenter comparison of intravesical hyaluronic acid/chondroitin sulfate with dimethyl sulfoxide shows that locally acting bladder therapies can be tested head-to-head rather than treated as interchangeable instillations [27]. Recurrent UTI provides another example of localized intravesical therapy and its practical scheduling constraints [28]. The causes differ, but the urinary tract repeatedly exposes the same practical issues of surface protection, local exposure, and recurrence.

Epithelial inflammatory memory offers another hypothesis for recurrence. Recent oral-barrier research suggests that epithelial stem or progenitor cells may retain transcriptional or epigenetic effects of prior inflammation [29,30]. Comparable urothelial memory has not been demonstrated in IC/BPS. For now, it remains a testable possibility: previous inflammation may leave the urothelium primed or slow to repair even after symptoms improve.

### 4.2. Innate Inflammation, Oxidative Stress, and the Immune–Vascular Interface

Barrier disruption and inflammation reinforce one another. Urinary solutes can activate urothelial and resident immune pathways, while reactive oxygen species further destabilize junctions, mitochondria, and regenerative programs [9,10]. ATP and neurotrophin signaling provide additional links between epithelial damage and pain amplification.

Comparable feedback is well described in IBD and oral mucositis [20,21,22,23,24,31]. IBD has connected these processes to objective mucosal-healing targets [32,33,34], while oral mucositis provides repair-oriented models [35,36]. Vascular inflammatory disease contributes a different perspective through endothelial dysfunction and immune–endothelial injury [37,38,39,40,41,42]. For IC/BPS, these comparisons argue for separating visible lesion inflammation from diffuse inflammatory activity, redox injury, and later neural amplification rather than treating all of them as one category.

Vasculitis is mainly an endothelial disorder, so the analogy has limits. It is still informative at the level of selective-barrier biology and mechanism-based classification. Studies of complement activation, autoantibody-mediated injury, and genetically defined vascular inflammation show how tissue patterns can support mechanistic stratification [37,38,39,40,41]. Behcet disease adds a multisystem example in which recurrent mucosal lesions coexist with vascular inflammation [42,43].

For IC/BPS, the useful extension is to study the urothelium together with its immune, neural, and vascular environment. This may be especially relevant in Hunner lesion disease. Vascular permeability or complement-related signals could eventually help identify a strongly inflammatory bladder phenotype [4,12]. The transferable principle is endotyping, not direct repurposing of vasculitis drugs.

### 4.3. Adaptive Immune Endotypes

IBD has moved from broad immunosuppression toward pathway-directed therapy. Current targets include TNF, IL-23-related pathways, integrins, JAK signaling, and S1P signaling [31,44]. IC/BPS should not copy that list. The relevant lesson is that a targeted immune therapy becomes more credible when the matching biological endotype can be identified before treatment.

Hunner lesion disease currently provides the clearest rationale for immune endotyping. Histopathology and B-cell findings support a distinct inflammatory phenotype [4,12]. Cyclosporine A has shown greater apparent utility in inflammatory or Hunner phenotypes than in unselected IC/BPS, although toxicity and limited evidence constrain routine use [2,6]. Intravesical interferon-alpha2b also produced a clinical signal compared with hyaluronic acid in a randomized trial [45]. These observations support immune-pathway testing in selected patients, but they do not define a universally responsive population.

IBD, eosinophilic esophagitis, and chronic rhinosinusitis show what pathway-directed treatment looks like when biological classification is more mature [31,44,46,47]. In eosinophilic esophagitis, a phase 3 trial of dupilumab improved histologic remission as well as dysphagia outcomes [47]. The connection between a defined pathway and an objective tissue response is the important point.

IC/BPS would benefit from the same discipline. Anti-NGF, interferon, B-cell-related targets, or future cytokine-directed agents should be linked to a pre-specified biological signature rather than grouped as interchangeable advanced therapies. This also makes a negative trial more informative because failure of the target can be separated from failure of patient selection.

### 4.4. Neuroimmune Sensitization and Dissociation Between Symptoms and Tissue Change

IC/BPS pain can persist after a mucosal insult has partly resolved [6,9]. Urothelial signaling and pelvic-floor nociception may continue to drive peripheral afferents, while central amplification becomes increasingly independent of the original tissue injury. Neural dysfunction therefore forms a therapeutic axis that overlaps with urothelial inflammation but is not identical to it. Botulinum toxin A and neuromodulation primarily aim to reduce abnormal signaling. Tanezumab offers a more pathway-specific example, although broader efficacy, safety, and responder selection remain unresolved [2,6,48].

Prospective work in refractory interstitial cystitis has shown that sacral neuromodulation can improve pain and voiding outcomes [49]. That improvement does not prove epithelial repair. This distinction matters in trial design. A patient with neurogenic-pain-dominant disease may improve markedly despite little change in mucosal biomarkers. Conversely, a regenerative treatment may improve barrier biology without fully reversing established central sensitization. These should be recognized as different forms of response rather than forced into one composite outcome.

### 4.5. Shared Architecture, Not Shared Etiology

Across chronic barrier diseases, the recurring similarity is architectural rather than cytokine-specific. Barrier failure can increase abnormal exposure [7,8,20,21]. Innate inflammation and oxidative stress may then amplify injury [9,20,21], while adaptive immunity can sustain disease in susceptible phenotypes [12]. Incomplete regeneration may leave tissue vulnerable to recurrence [29,30,50].

Other processes modify that core architecture. Broader epithelial-barrier literature describes similar cross-organ patterns [51,52,53,54,55]. Neural sensitization can maintain symptoms even when visible tissue injury improves [49]. Microvascular inflammation may sustain selected phenotypes [37,38,39,40], and dysbiosis can alter local or systemic exposure. These factors are better viewed as possible amplifiers than as universal initiating events.

Applied to IC/BPS, this logic favors mechanism-based sequencing. The dominant phenotype should be defined first. Tissue or barrier activity can then be assessed when it is likely to change interpretation. Noninvasive biomarkers may support that assessment, but they should not stand alone. Treatment selection and follow-up should return to the same biological hypothesis. Comparator diseases help organize this process without proving that their organ-specific therapies will work in IC/BPS. The shared mechanistic axes are summarized in Table 1.

## 5. Therapeutic Migration into IC/BPS: From Shared Mechanism to Bladder-Directed Therapy

Cross-organ comparison becomes most persuasive when a shared mechanism can be followed into an actual therapeutic migration. Several IC/BPS interventions matured in other specialties before being tested in the bladder. That history does not imply a common etiology. It asks a narrower question: if the same treatable process is active in a bladder endotype, does an intervention that modifies it elsewhere deserve bladder-specific testing? Failed migrations are informative too. They can reveal problems in patient selection, tissue context, delivery, or outcome definition.

### 5.1. Barrier Restoration and Local Anti-Inflammatory Treatment

Barrier restoration is the most direct therapeutic extension of the urothelial model. Intravesical hyaluronic acid, chondroitin sulfate, and heparin-containing regimens seek to reduce exposure of a vulnerable surface to urine [2,6,27]. Oral pentosan polysulfate (PPS) arose from a related concept, but its mechanism and clinical benefit remain uncertain. Long-term PPS exposure has been associated with pigmentary maculopathy; the product label calls for ophthalmic history, retinal assessment including optical coherence tomography and autofluorescence within six months of initiation, and periodic review during long-term use [57]. Its weak anticoagulant activity also warrants attention to bleeding risk. Intravesical agents can cause pain, infection, or catheter-related injury, so adverse events and procedural burden belong in any study of local protection. Biological heterogeneity is only one possible source of variable treatment effects, alongside differences in dose, exposure, and study quality.

IBD suggests a useful way to test this idea. Prospective studies show that deeper endoscopic or histologic healing is associated with better subsequent disease control [58,59]. Treat-to-target frameworks therefore place objective tissue outcomes alongside symptoms [60,61]. An analogous IC/BPS program could ask whether a bladder-protective treatment changes urothelial differentiation or a permeability surrogate, then examine whether that biological change predicts recurrence. Symptom scores would remain important, but they would no longer carry the entire interpretation.

Current IC/BPS therapies already show why mechanism matters. Dimethyl sulfoxide is a broad intravesical anti-inflammatory and analgesic treatment with variable evidence [62]. Triamcinolone injection and fulguration, in contrast, are directed at visibly inflamed Hunner lesions and are supported in guideline-based management [2,6]. Eosinophilic esophagitis provides a useful external analogy: a randomized trial of budesonide orodispersible tablets achieved clinical and histologic remission by concentrating corticosteroid exposure at the diseased mucosa [63]. That supports local, target-relevant delivery. It does not justify generalized steroid treatment for non-Hunner IC/BPS. Experimental antioxidant or inflammasome-directed approaches should likewise include biological measures that confirm target engagement [9,10].

### 5.2. Regenerative Migration: PRP, Stem Cells, Amniotic Tissue, and Shock-Wave Therapy

Platelet-rich plasma (PRP) is a representative regenerative migration. Its development across orthopedics and wound care is supported by randomized trials in conditions such as knee osteoarthritis and chronic diabetic foot ulcers [25,64,65]. In those settings, autologous platelet concentrates are used as a source of repair-associated mediators.

The move into IC/BPS rests on a different but related hypothesis: some patients may have a repair-deficient urothelium in which incomplete regeneration sustains symptoms. Primary IC/BPS studies have reported symptom improvement after repeated intravesical PRP, together with changes in urinary functional proteins and other clinical outcomes [66,67]. Protocols and objective healing measures remain heterogeneous. The more informative next question is whether a repair-deficient endotype predicts response.

Mesenchymal stem-cell (MSC) therapy represents a further regenerative migration. Cell-based and extracellular-vesicle strategies have also been studied in inflammatory bowel disease [68]; this does not establish their efficacy in IC/BPS. A single-center phase I/IIa trial enrolled 22 patients with refractory Hunner-type disease: six received open-label dose escalation in phase I, and 12 received MR-MC-01 versus four saline-placebo injections in randomized phase IIa [69]. At six months, the treatment and placebo groups changed by −5.50 versus −3.00 points on the ICQ (between-group *p* = 0.56), and by −4.75 versus −2.25 points on the PUF (*p* = 0.18). The reported nocturnal-voiding contrast favored treatment, whereas moderate or marked global improvement was 5/12 versus 1/4; no inferential comparison for that global proportion was reported. The paper also states that 8/12 treated patients had reduced or resolved Hunner lesions at six months. However, its patient-level outcome table does not establish that count: three entries are missing at six months, two explicitly read “lesion resolved”, six read “no change”, and one reads “lesion formation”. The table does not specify whether “no change” refers to baseline or the preceding visit, and it does not provide patient-level lesion results for the four placebo recipients. We therefore cannot verify the eight-of-twelve six-month claim or infer comparative structural recovery from it. These findings warrant a prespecified, blinded lesion assessment in a larger trial, alongside symptom and safety outcomes.

Amniotic tissue provides another regenerative example. Amniotic membrane is established in ocular-surface reconstruction and has properties that support re-epithelialization and modulation of inflammation [70]. Small, uncontrolled studies later tested micronized amniotic products in refractory IC/BPS. An initial 10-patient report described short-term improvement, and a 25-patient follow-up suggested that some responses persisted to 6 months [71,72]. These findings are translational signals, not confirmation of efficacy.

Low-energy extracorporeal shock-wave therapy (LESW) moved from musculoskeletal pain and tissue-repair research into lower urinary tract disorders [73,74]. A randomized trial assigned 51 adults receiving LESW to BoNT-A or saline instillation, with eight weeks of follow-up [74]. Because both arms received LESW, that comparison tests the added instillation rather than the effect of LESW against sham exposure. Clinical or urinary biomarker changes alone do not demonstrate epithelial repair. For both LESW and amniotic products, a tissue-repair claim would need a validated, prespecified structural or functional assessment.

### 5.3. Neural and Immune Migration: Testing Endotype Relevance

Botulinum toxin A (BoNT-A) and neuromodulation illustrate migration from neurology and chronic-pain therapeutics into bladder disorders. Their rationale rests on abnormal afferent signaling and network excitability [49,56]. Because the intended effect is neural, tissue-healing endpoints should not be used as the sole measure of success. Immune-directed therapy raises a different problem. Cyclosporine provides broad immunomodulation in selected IC/BPS pathways [2,6], while intravesical interferon represents a more targeted strategy [45]. Interferon-alpha2b showed a clinical signal versus hyaluronic acid in a randomized trial [45]. Tanezumab produced a symptom signal through NGF blockade [48]. These results support pathway testing, but not a universal advanced-therapy pathway for unselected IC/BPS.

An instructive negative migration is intravesical bacillus Calmette–Guérin (BCG), adapted from bladder-cancer immunotherapy. In a multicenter double-blind trial, 265 patients with treatment-refractory IC were randomized to BCG or placebo; global response at 34 weeks was 21% versus 12% (*p* = 0.062) [75]. The primary comparison did not meet conventional statistical significance. Smaller positive signals cannot override this larger result. The failure might reflect target biology, study population, dosing, or chance, but none of these explanations was established prospectively. BCG should not be rehabilitated for IC/BPS solely by postulating an unmeasured endotype, especially given its capacity to provoke cystitis and rare systemic infection.

### 5.4. Microbiome Migration: From FMT to Urinary Microbiota Transplantation

Microbiome therapy is the most literal migration considered here. Fecal microbiota transplantation (FMT) first gained a clinical role in recurrent Clostridioides difficile infection. This prompted interest in whether ecosystem-level interventions might also affect the urinary tract. In a 2025 proof-of-concept study, 22 postmenopausal women with recurrent urinary tract infection received FMT and experienced fewer urinary infectious episodes over six months [76]. That uncontrolled study does not establish relevance to IC/BPS. It does, however, give a reason to study whether a gut-directed intervention can influence urinary outcomes.

In IC/BPS, biological plausibility has advanced more quickly than therapeutic evidence. Multi-omics work in Hunner-type disease linked gut microbial composition and bile-acid metabolism to urothelial TLR3 activation and barrier injury [19]. A 2026 longitudinal urobiome analysis did not find a broad microbial signature that cleanly separated pain phenotypes, although more nuanced taxon-level associations with symptom localization were observed [77]. The signal is biologically interesting, but heterogeneity remains substantial.

Direct urinary microbiota transplantation (UMT) is much earlier in development. A three-patient retrospective report described short-term symptoms and individual follow-up, without a control group or an established efficacy endpoint [78]. A 2026 consensus addresses procedural standardization but does not validate UMT efficacy in IC/BPS [79]. Future studies need an ethics-approved protocol and regulatory review appropriate to the jurisdiction, written informed consent explaining infectious and unknown long-term risks, prospective adverse-event reporting, and an independent safety plan. Donors should be screened by medical and exposure history and by validated testing for urinary pathogens, blood-borne infections, and relevant multidrug-resistant organisms; products need traceable collection, processing, quarantine/release rules, and contamination controls. Infections transmitted by fecal microbiota products show why analogous vigilance is necessary, though fecal rules cannot simply be copied to urine [80]. Changes in microbiome composition or engraftment would establish neither symptomatic efficacy nor safety. UMT remains a research intervention.

### 5.5. A Migration Filter for Future Therapies

The proposed migration filter is a way to rank research questions, not a clinical eligibility rule. Investigators should assess whether a plausible target can be demonstrated in bladder tissue or a specified subgroup and whether that subgroup can be identified reproducibly before treatment. A conserved mechanism alone is insufficient: the exposure, biological context, and intervention-related risks may differ across organs.

A candidate study should prespecify symptom outcomes, a feasible measure of the proposed target where available, and intervention-specific safety monitoring. Local procedures bring infection, bleeding, and anesthesia risks; systemic immunomodulation needs toxicity surveillance; and cells and living microbial products add manufacturing, immune, transmission, and long-term uncertainties. A controlled trial can establish symptomatic efficacy without validating the mechanism. Conversely, a biomarker shift is evidence of possible target engagement, not proof of clinical benefit or disease modification. These distinctions prevent a failed trial from being explained away by endotype mismatch without direct supporting data.

For now, the migration filter should be treated as a research tool, not a validated clinical decision rule. Its value will depend on reproducible endotypes and practical measures of target engagement. If those pieces cannot be measured prospectively, the filter remains conceptual. Representative migrations and their evidence maturity are summarized in Table 2.

## 6. Why Rational Therapies Produce Inconsistent Results: Testing Endotype Mismatch

### 6.1. The Current Gap Between Diagnosis and Biology

IC/BPS diagnosis remains clinical and exclusionary. Treatment response is also judged largely through symptoms and voiding outcomes. Those measures matter because they capture the lived disease, but they do not show whether urothelial injury has resolved or whether the intended biological pathway has changed. Similar symptom scores can therefore arise from different disease states.

Therapeutic selection remains largely empirical. Guidelines and broad syntheses do not identify one intervention that improves every outcome in every patient [2,5,6]. DMSO and PRP studies illustrate how uneven protocols and comparators complicate interpretation [62,66,67]. Endotype mismatch is a plausible explanation for some inconsistent results, but inadequate exposure, small samples, measurement error, and genuine lack of efficacy must also be considered. No current biomarker panel has prospectively validated these proposed categories for choosing IC/BPS treatment.

There is also no validated objective recovery target for IC/BPS. Cystoscopy remains essential for identifying Hunner lesions and for selected diagnostic questions, but the field lacks a standardized measure that links symptoms to tissue activity and barrier recovery. IBD has mucosal-healing targets; IC/BPS has no accepted equivalent. This gap motivates the translational framework proposed here.

### 6.2. From Phenotype to Endotype

A barrier-oriented research strategy should refine the clinical diagnosis rather than replace it. Current consensus supports separating Hunner lesion disease and distinguishing bladder-centric from systemic presentations [1,2,3]. Biomarker work suggests that composite signatures may add information beyond single analytes [14,15,16,17]. A future work-up could therefore combine clinical phenotype with a limited set of examination and biomarker measures chosen for a specific treatment question.

For research purposes, six overlapping candidate patterns may help organize trials. A barrier-dominant hypothesis concerns currently demonstrable surface or permeability dysfunction, with symptom timing linked to bladder filling; a repair-deficient hypothesis would require evidence of delayed restitution or persistent epithelial injury on serial assessment after active inflammation has diminished. Neither has an accepted assay or threshold. Hunner lesions provide an observable inflammatory feature, but proposed cytokine and BAFF/APRIL cutoffs are unvalidated. Pelvic-floor tenderness is an examination finding and does not demonstrate afferent sensitization or a central neural mechanism. Neural sensitization remains a separate hypothesis requiring direct evidence. Widespread pain and comorbid symptoms describe clinical distribution rather than a proven molecular endotype. These patterns can overlap and should not be treated as diagnostic labels.

Chronic rhinosinusitis shows how a field can move beyond descriptive phenotypes. Histology and biomarker-based approaches are increasingly used to define inflammatory endotypes, with the longer-term aim of improving selection for targeted biologics [46]. IC/BPS need not copy those immune labels. A useful bladder endotype should be reproducible, clinically measurable, linked to disease biology, and capable of changing a treatment decision or interpretation. Depending on the question, Hunner status could be combined with a urinary panel or a tissue feature rather than with an exhaustive battery of tests.

The corresponding study hypotheses differ by putative mechanism: surface protection in patients with a reproducible barrier measure, repair interventions where serial injury and restitution can be documented, lesion-directed approaches in verified Hunner disease, and pain-focused care when pelvic-floor or widespread symptoms predominate [2,6,66,67]. These are candidates for stratified trials, not rules for selecting or withholding an intervention in current practice. Table 3 makes the proposed measurements and gaps explicit.

### 6.3. Mechanism-Matched Sequencing Instead of an Empiric Treatment Ladder

These mechanisms suggest several testable treatment sequences rather than a universal ladder. A trial might stratify by Hunner status and prespecified bladder or pain features before evaluating whether a mechanism-matched intervention improves outcomes. Combinations need a clear rationale and a comparator able to isolate any added benefit. Current evidence does not justify assigning every patient a dominant molecular axis or using the proposed sequence as a clinical algorithm.

A 122-patient observational analysis reported within-group symptom changes after PRP and BoNT-A and different urinary-marker trajectories after four therapies [81]. The fact that some within-group tests reached significance while others did not does not establish that PRP or BoNT-A outperformed hyaluronic acid or LESW. That requires a prespecified between-group contrast with appropriate adjustment for baseline differences. A retrospective multimodal-therapy study is likewise susceptible to selection and confounding [82]. Such findings can generate candidate stratification variables, but prospective controlled comparisons are needed before inferring comparative efficacy or a successful endotype match.

## 7. Urothelial Healing as a Multidimensional Treatment Target

The most transferable lesson from IBD is a treatment architecture rather than a specific drug. Treat-to-target programs separate symptom improvement from objective disease control. STRIDE-II formalized this by linking early clinical response to later reassessment of biological activity and healing [61]. Prospective IBD studies likewise show that deeper endoscopic or histologic healing can carry prognostic information [58,59].

IC/BPS has no validated counterpart. A reasonable adaptation is to measure symptoms and urothelial recovery as related but distinct outcomes. That approach is most relevant to bladder-centered disease with active barrier injury, inflammation, or impaired repair. It should not be imposed on predominantly neurogenic or systemic-pain phenotypes.

We use “urothelial healing” as a research construct, not a validated IC/BPS endpoint. Clinical response denotes meaningful change in symptoms and quality of life; it does not imply tissue restoration. An endoscopic response describes visible mucosal change, most clearly when a Hunner lesion is reassessed with consistent methods. Neither measure alone demonstrates restored urothelial function.

Molecular target engagement asks whether a prespecified pathway changed in the expected direction. A functional barrier response requires evidence of improved urothelial integrity or permeability. “Healing” should be reserved for a validated tissue-recovery measure. Disease modification is a stronger claim and would additionally require durable clinical outcomes together with credible evidence that the disease process has been altered. No IC/BPS assay or threshold currently satisfies all these conditions.

The methodological value of this construct is simple: symptomatic relief and biological repair are not the same event. Pain can improve while barrier abnormalities persist. Epithelial repair can occur while a systemic pain phenotype remains active. Inflammation may also fall without correcting a regenerative defect. Multidomain assessment makes those discordant responses visible.

Validation should start with prognosis. A candidate urothelial-healing measure must be reproducible and responsive to treatment. More importantly, it should add information that matters clinically, such as prediction of durable remission or fewer flares. A measure that does not improve decisions beyond standard symptom assessment is unlikely to justify routine use. Candidate domains and measures are summarized in Table 4.

## 8. A Proposed Treat-to-Target Framework for IC/BPS

### 8.1. Define the Dominant Biology and a Pre-Specified Target

Treat-to-target is a borrowed research architecture rather than current IC/BPS standard care. STRIDE-II in IBD links symptom response with repeated objective assessment of bowel activity [61]. In IC/BPS, a trial could prespecify a clinical outcome and, where appropriate, a separately analyzed lesion or barrier endpoint. The direction, time point, reproducibility, and prognostic value of any proposed tissue endpoint need validation before it can serve as a target. Improvement in an IBD endpoint does not validate a urothelial-healing endpoint.

Prospective trials should stratify patients before randomization. Hunner status and the distinction between bladder-centric and systemic presentations should be recorded at minimum [3]. Additional measures can be added when they are relevant to the mechanism under study. The aim is not to create a maximal testing panel. It is to distinguish a biologically ineffective treatment from a potentially useful treatment tested in the wrong population.

### 8.2. Reassess Symptoms and Biological Activity Separately

Objective barrier assays need technical and clinical validation. Exfoliated urothelial cells, organoids, urinary proteins, and permeability surrogates could be tested for reproducibility, change over time, and added prognostic value [14,15]. Proposed panels lack accepted thresholds and cannot currently determine whether a patient should continue, switch, or stop treatment. In Hunner disease, repeat cystoscopy can document lesion status when clinically indicated or prospectively justified, but lesion change still should not be equated automatically with regenerated epithelium [4,12].

### 8.3. Treat Recurrence as a Mechanistic Outcome

Recurrence is a clinically meaningful outcome, but fewer flares alone cannot show tissue repair or disease modification. The inflammatory-memory literature suggests a straightforward experimental design [30]. Urothelial cells could be sampled during active disease and again after the response. A third sample at relapse would then allow investigators to ask whether prior inflammation left a stable transcriptional or epigenetic imprint. This is an untested bladder hypothesis; sampling burden and prognostic value must be justified before it becomes a trial target.

### 8.4. Treat Local Exposure as a Modifier, Not the Disease Model

For intravesical therapy, delivery performance matters when it can confound biological interpretation. A drug may fail because urinary dilution prevents adequate exposure, not because the target is irrelevant. The reverse is also true: prolonged residence time does not establish efficacy. Exposure and procedural burden are therefore useful secondary variables, while mechanism-specific target engagement should remain central [83,84,85]. Table 5 summarizes the proposed sequence.

## 9. Research Priorities and Limits of Cross-Organ Extrapolation

### 9.1. Priority Research Directions

Several research priorities follow from this framework. IC/BPS endotypes need to become reproducible and treatment-predictive rather than merely descriptive. A biomarker panel or tissue assay is useful only if it changes selection, prognosis, or interpretation. Trials of regenerative, immune, neural, and microbiome therapies should also enrich for the biological state that the intervention is designed to modify.

Early uncontrolled work can establish feasibility and generate hypotheses. Adequately controlled trials can establish symptomatic efficacy without proving a biological mechanism. Target engagement requires reliable, prespecified biological measures; urothelial regeneration requires direct and validated structural or functional evidence. A disease-modification claim would additionally need durable, clinically meaningful benefit supported by coherent tissue-level evidence. Safety monitoring must match the intervention, including retinal surveillance with long-term PPS and infectious-disease safeguards for living microbial products [57,80].

### 9.2. Limits of Extrapolation

Cross-organ comparison is useful only when biological differences remain explicit. The intestinal mucosa cannot be mapped directly onto the bladder because its microbial ecosystem and mucus architecture are different. Oral mucositis and chronic wounds are primarily injury-repair models, not idiopathic chronic pain disorders. Delivery platforms developed for the eye, esophagus, or vagina also face physiological constraints that differ from urine-exposed urothelium.

Other analogies have different limitations. Chronic rhinosinusitis has clinically actionable inflammatory endotypes that IC/BPS does not yet possess. Radiation cystitis and recurrent UTI share the organ but not the initiating cause. Vasculitis centers on endothelium rather than epithelium. The comparisons made here therefore concern mechanisms and study design, not common etiology or direct drug interchangeability.

The comparator diseases do not contribute equally. IBD offers the closest structural analogy for the relation among barrier injury, inflammation, and tissue healing. Eosinophilic esophagitis and chronic rhinosinusitis are more useful for thinking about endotype-guided treatment. Oral mucositis and chronic wounds mainly inform injury and repair. Same-organ conditions such as radiation cystitis or recurrent UTI are valuable for testing whether a proposed principle still makes sense in the urinary environment.

What transfers most reliably is a way to frame falsifiable studies. Objective biological measures can complement symptoms when they are reliable and linked to a prespecified mechanism. Neither a failed migration such as BCG nor an early positive signal can be attributed automatically to endotype selection without prospective evidence [75]. Reassessment may improve treatment decisions, but the proposed rules must first be compared with usual care.

## 10. Conclusions

Urothelial barrier dysfunction remains useful for a substantial subset of IC/BPS, but it is more informative as part of a network than as a single-cause theory. Barrier failure can interact with inflammation, altered sensory signaling, microvascular change, and defective regeneration. The balance among these processes is likely to differ across phenotypes.

Comparator diseases clarify different parts of that network. IBD shows how tissue healing can be linked to objective monitoring, while other fields contribute models of endotyping, repair, or neural modulation. Their main contribution is methodological. They help define better questions for IC/BPS rather than supplying a ready-made treatment list.

Therapeutic migration offers a way to choose and test bladder-relevant hypotheses. Its proposed filter, candidate endotypes, and urothelial-healing domains remain investigational. Controlled trials can demonstrate clinical response or symptomatic efficacy even if the biological mechanism is unresolved. A marker change may support target engagement but does not establish regeneration; fewer flares may indicate durable benefit but do not independently prove disease modification. Structural recovery requires a reliable, prespecified tissue measure, and any disease-modification claim needs durable clinical outcomes alongside credible biological evidence. Safety and negative trials, including intravesical BCG, should inform which migrations proceed. Until these measures and selection strategies are validated, clinical decisions should continue to rely on established evidence and patient-centered assessment [2,75].

## Figures and Tables

**Table 1:** Shared mechanistic axes across barrier diseases and their relevance to IC/BPS.

Shared Axis	Evidence from Comparator Diseases	IC/BPS-Centered Interpretation	Therapeutic/Measurable Implication
Structural barrier failure	Tight-junction, mucus or epithelial disruption in IBD/oral disease; endothelial junction/glycocalyx dysfunction in vascular disease	GAG/umbrella-cell/uroplakin and junctional defects may increase exposure to urinary irritants	Test GAG/barrier-directed therapy in barrier-active patients; monitor cystoscopic phenotype, uroplakins, junctional proteins, and permeability surrogates.
Innate inflammation/oxidative stress	ROS, innate sensing and inflammatory feed-forward loops disrupt barrier repair in mucosal and vascular disorders	Urothelial ROS and innate signaling may sustain inflammation and amplify ATP/NGF/TRP-related nociception	Distinguish broad local anti-inflammatory therapy from pathway-specific strategies; include inflammatory/redox target-engagement markers
Adaptive immune dysregulation	Targeted therapy in IBD, eosinophilic esophagitis, and chronic rhinosinusitis is increasingly linked to pathway-defined inflammatory phenotypes or endotypes [31,44,46,47].	Clonal B cells and APRIL/BAFF are especially relevant to Hunner lesion disease; other immune endotypes remain to be defined [4,12].	Use Hunner status and immune profiling to enrich future cyclosporine, interferon, or targeted-immunotherapy studies [2,6,45].
Neuroimmune sensitization	Prospective IC/BPS neuromodulation studies show symptom improvement; tissue repair is not established [49,56].	Peripheral afferent and central amplification can uncouple symptoms from visible bladder injury	In stratified studies, test symptom and functional outcomes for neural interventions separately from urothelial-repair endpoints.
Defective repair/inflammatory memory	Failed epithelial restitution and persistent epithelial reprogramming can promote recurrence [29,30,50].	Repair-deficient states may sustain flares; inflammatory memory is a testable bladder hypothesis	Evaluate platelet-rich plasma and other regenerative strategies with repair markers, durability, and flare recurrence rather than pain alone.
Microvascular dysfunction	Endothelial activation, vascular leak, and leukocyte trafficking sustain tissue inflammation [37,38,39,40].	Potentially important in highly inflamed or Hunner phenotypes [4,12].	Study endothelial/vascular markers and spatial immune–vascular features; do not infer direct benefit from vasculitis drugs

**Table 2:** Representative therapeutic migrations into IC/BPS: migration rationale, study design, findings, and interpretive limitations.

Strategy	Field of Origin/Maturation	Conserved Target Supporting Migration	Study Design, Sample, Comparator, and Follow-Up	Clinical/Tissue Findings	Limitations and Safety
PRP [66,67]	Orthopedics, sports medicine, and chronic-wound care	Platelet-derived growth factors and repair-associated mediators used in orthopedic and wound-healing settings may be relevant to a repair-deficient urothelium [25,64,65,66,67].	Two IC/BPS cohorts: [67]: specifically enrolled non-Hunner patients; [66]: prospective repeated-injection cohort, *n* = 40; no concurrent control; four monthly injections with 1- and 3-month post-course assessments. [67]: single-center cohort, *n* = 98; four monthly injections; outcomes assessed 3 months after treatment; no concurrent control.	Study [66] reported symptom improvement and changes in urinary functional proteins, without establishing tissue restoration. In [67], 54/98 (55.1%) had GRA ≥ 2 at 3 months after treatment; symptoms and maximum bladder capacity improved from baseline.	Uncontrolled designs limit causal inference; placebo and procedure effects remain possible. PRP preparation and response assessment vary, and neither study used a validated urothelial-healing endpoint.
hESC-derived MSCs [69]	Regenerative medicine and inflammatory/wound-repair research	Paracrine immunomodulation and regenerative signaling could support recovery of injured bladder tissue [68,69].	Single-center phase I/IIa; total *n* = 22; randomized phase IIa *n* = 12 active and *n* = 4 placebo; submucosal cells versus saline; 1, 3, and 6 months.	At 6 months, between-arm ICQ *p* = 0.56 and PUF *p* = 0.18; nocturia favored cells. The reported 8/12 lesion responses cannot be reconstructed from the patient-level Table 6.	Tiny placebo arm; three six-month lesion entries missing; ambiguous “no change”; no patient-level placebo lesion table; long-term safety remains uncertain.
Amniotic product [71,72]	Ocular-surface reconstruction and wound healing	Anti-inflammatory, anti-scarring, matrix-support, and epithelial-healing properties provide the rationale for migration from ocular-surface repair [70,71,72].	Consecutive single-arm series; *n* = 10 initially and *n* = 25 in the six-month follow-up report; no concurrent comparator; follow-up to 3 and 6 months.	Some symptom improvement was reported; seven patients requested repeat injection at month 4 [72].	Retreatment obscures durability; no sham control or direct barrier-repair measure; product standardization remains an issue.
LESW [74]	Musculoskeletal pain and tissue-repair research	Mechanical stimulation may modulate tissue-repair, inflammatory, and pain-related pathways that intersect with IC/BPS biology [73,74].	Randomized double-blind trial, *n* = 51; all participants received four LESW sessions; BoNT-A instillation (*n* = 25) versus saline (*n* = 26); 8 weeks.	Clinical and urinary biomarker outcomes were reported for the added BoNT-A question.	No sham-LESW arm, so LESW efficacy and epithelial regeneration cannot be inferred; follow-up was short.
Neuromodulation [49]	Neurology and chronic-pain medicine	Reduction in afferent hyperexcitability and neural amplification is relevant to pain-dominant phenotypes [49,56].	Prospective cohort; *n* = 25 tested and 17 implanted; no sham control; mean follow-up 14 months after implantation.	Pain and voiding outcomes improved among selected implanted patients.	Responder selection and uncontrolled design limit inference; neural symptom benefit is not evidence of epithelial repair; device and procedure risks remain relevant.
Intravesical interferon [45]	Antiviral and immune-directed therapy	Local immune modulation is a plausible migrated strategy for inflammatory IC/BPS, but the relevant responder pathway has not been validated [4,12,45].	Single-center double-masked randomized trial, *n* = 52 women; interferon *n* = 26 versus hyaluronic acid *n* = 26; 6 months.	Pain and ICSI/ICPI favored interferon at month 6.	Active comparator and single-center design; antiviral or immune target engagement was not confirmed; replication is required.
UMT [78,79]	Gastroenterology and recurrent-infection microbiome therapy	Microbial ecosystems may influence urinary immune tone, metabolites, and epithelial signaling, although IC/BPS microbiome signatures remain heterogeneous [19,76,77,78,79].	Retrospective uncontrolled report, *n* = 3 women; no comparator; individual cases followed for 13, 15, and 20 months with assessments at differing time points.	Exploratory symptom and microbiota observations were reported.	Very small retrospective series; screening standards and product reproducibility require validation; infection transmission and long-term safety remain uncertain.
BCG, negative example [75]	Bladder-cancer immunotherapy	BCG was migrated on the premise that local immune activation might modify refractory IC/BPS; the larger trial tested whether that premise translated into clinical benefit [75].	Multicenter double-blind randomized trial, *n* = 265; BCG versus placebo; six weekly instillations; 34 weeks.	Global response was 21% versus 12% (*p* = 0.062); the primary comparison did not reach conventional statistical significance.	Small positive studies do not overcome this trial; inflammatory and infectious risks remain, and there is no basis to assume an unmeasured responder endotype.

Note: The rows describe selected studies rather than pooled effects. The two PRP reports are shown separately within one row because they represent different cohorts (*n* = 40 [66] and *n* = 98 [67]). Phase I participants are excluded from the randomized MSC comparison. Abbreviations: BCG, bacillus Calmette–Guérin; BoNT-A, botulinum toxin A; hESC, human embryonic stem cell; LESW, low-energy extracorporeal shock-wave therapy; MSC, mesenchymal stem cell; PRP, platelet-rich plasma; UMT, urinary microbiota transplantation.

**Table 3:** Candidate IC/BPS patterns and trial hypotheses. These categories and indicators are unvalidated for treatment selection.

Candidate Pattern	What Would Distinguish It in a Study	Intervention Hypothesis to Test	Validation Gap and Research Endpoint
Barrier-active	Bladder-centered filling-related symptoms plus reproducible evidence of current surface injury or permeability; no validated IC/BPS cutoff.	Test protective instillation against an appropriate comparator [2,62].	Standardize barrier assay and threshold; symptoms plus longitudinal integrity measure.
Repair-deficient	Serial evidence that epithelial damage persists or restitution is delayed after inflammation subsides; a low repair marker alone is insufficient.	Test PRP or a defined regenerative product in an enriched, controlled cohort [66,67].	No validated restitution assay or cutoff; repeat tissue/functional assessment and durability.
Immune–inflammatory	Cystoscopically confirmed Hunner lesions are observable; cytokine and BAFF/APRIL profiles remain investigational.	Test lesion-directed or immune-pathway intervention with prespecified activity measures [2,4,12,45].	No validated molecular threshold; compare symptoms and blinded lesion assessment.
Pelvic-floor-associated	Pelvic-floor tenderness or trigger points are observable examination findings; they identify a clinical myofascial pattern but do not demonstrate afferent or central sensitization.	Test pelvic-floor-directed therapy in patients selected by prespecified examination findings.	Standardize examination methods and measure pain, function, and bladder-specific symptoms; do not infer a neural mechanism from myofascial findings alone.
Neural-sensitization hypothesis	Afferent or central sensitization requires direct neural or neurophysiological evidence; symptoms alone and pelvic-floor tenderness are insufficient, and no validated IC/BPS neural assay exists.	Test neuromodulation, BoNT-A, or other neural-directed strategies in prospectively defined symptom strata, with neural measures included when feasible [49,56].	No validated neural biomarker or cutoff for treatment selection; assess clinical pain/function separately from directly measured neural target engagement.
Systemic pain overlap	Widespread pain and multisystem symptoms define a clinical pattern, not a demonstrated bladder endotype.	Study integrated pain and comorbidity management without assuming absent bladder disease.	Measure pain distribution, sleep, quality of life, and bladder-specific symptoms.

**Table 4:** Proposed research domains for urothelial healing.

Domain	Candidate Measures	Current Status	Key Validation Question
Clinical response	Pain VAS/NRS; ICSI/ICPI; urgency/frequency/nocturia; global response; quality of life	Established, but not tissue-specific	Does symptom improvement track objective barrier recovery?
Endoscopic lesion response	Hunner lesion status; erythema/bleeding; standardized mucosal photography	Hunner identification is established; longitudinal lesion scoring and predictive thresholds are not.	Can reproducible endoscopic change predict durable response or recurrence?
Molecular target engagement	Inflammatory cytokines; matrix metalloproteinase-9 (MMP-9); BAFF/APRIL; oxidative markers; NGF; repair transcripts/proteins	Exploratory panels; no validated IC/BPS threshold or surrogate endpoint.	Can a composite panel define low-activity or repair-restored states?
Functional barrier response	Uroplakins (UPKs), ZO-1, E-cadherin, claudins; permeability surrogates; exfoliated-cell or organoid assays	Research-stage assays without validated clinical cutoffs.	Which feasible test best predicts prognosis or response?

**Table 5:** Prospective questions for testing a treat-to-target model in IC/BPS. These are hypotheses for prospective evaluation, not validated clinical continuation or switching rules.

Study Stage	Question to Test	Candidate Measures	Prespecified Comparison or Hypothesis
Baseline stratification	Do recorded phenotypes modify treatment effect?	Hunner status; bladder-centered/systemic features; pelvic-floor examination; candidate markers.	Compare treatment effects across prespecified strata; do not require an unvalidated panel for care.
Early assessment	Does the intended pathway change, and does this predict later benefit?	A reproducible treatment-specific marker where available; symptoms and harms.	Test target engagement as a separate outcome; do not use it alone to determine continuation, switching, or clinical efficacy.
Response domains	Do symptoms, visible lesions, and biological measures agree?	Pain, ICSI/ICPI, quality of life; cystoscopy when justified; candidate barrier assays.	Analyze clinical efficacy separately from target engagement and tissue change.
Adaptive strategy	Does reassessment improve outcomes after partial response?	Repeated clinical phenotype and selected measures with known measurement properties.	Test a prespecified adaptive strategy against usual care before any continuation or switching rule is proposed for clinical use.
Durability	Does an early signal predict meaningful longer-term outcomes?	Flares, repeat treatment, lesion recurrence, adverse events, patient function.	Fewer flares alone do not prove regeneration or disease modification.

## Data Availability

No new data were created or analyzed in this study. Data sharing is not applicable to this article.
